# Measuring Adolescent Self-Awareness and Accuracy Using a Performance-Based Assessment and Parental Report

**DOI:** 10.3389/fpubh.2018.00015

**Published:** 2018-02-02

**Authors:** Sharon Zlotnik, Joan Toglia

**Affiliations:** ^1^Laboratory for Innovation in Rehabilitation Technology Israel Center for Research Excellence: LINKS – Learning in a NetworKed Society, Department of Occupational Therapy, University of Haifa, Haifa, Israel; ^2^School of Health and Natural Sciences, Mercy College, Dobbs Ferry, NY, United States

**Keywords:** adolescents, self -awareness, executive functions, weekly calendar planning activity, behavior rating inventory of executive function

## Abstract

**Aim:**

The aim of this study was to assess awareness of performance and performance accuracy for a task that requires executive functions (EF), among healthy adolescents and to compare their performance to their parent’s ratings.

**Method:**

Participants: 109 healthy adolescents (mean age 15.2 ± 1.86 years) completed the Weekly Calendar Planning Activity (WCPA). The discrepancy between self-estimated and actual performance was used to measure the level of awareness. The participants were divided into high and low accuracy groups according to the WCPA accuracy median score. The participants were also divided into high and low awareness groups. A comparison was conducted between groups using WCPA performance and parent ratings on the Behavior Rating Inventory of Executive Function (BRIEF).

**Results:**

Higher awareness was associated with better EF performance. Participants with high accuracy scores were more likely to show high awareness of performance as compared to participants with low accuracy scores. The high accuracy group had better parental ratings of EF, higher efficiency, followed more rules, and were more aware of their WCPA performance.

**Conclusion:**

Our results highlight the important contribution that self-awareness of performance may have on the individual’s function. Assessing the level of awareness and providing metacognitive training techniques for those adolescents who are less aware, could support their performance.

## Introduction

Self-awareness is a metacognitive process that is required to achieve successful outcomes in daily life. In clinical settings, awareness training is aimed toward enhancing self-understanding of strengths and weaknesses so that the person is then able to set realistic goals and improve occupational performance (1, 2).

According to Roebers (3), current conceptualizations of metacognition distinguish between (a) declarative metacognitive knowledge; the knowledge about cognition and learning processes, (b) procedural metacognition; the subjective assessments of ongoing cognitive activities, and (c) metacognitive control; the regulation of current cognitive activities. Metacognitive experiences made during learning and remembering (procedural metacognition) will lead back to changes in metacognitive knowledge.

Self-awareness reflects this dynamic relationship between the person’s knowledge, his beliefs, the task demands, and the context of the situation. Awareness of performance is the person’s ability to self-monitor, recognize errors, and correct them during a task. It influences the ability to select appropriate task strategies, which in turn, can improve performance. A retrospective performance judgment immediately following the task, compared with actual performance, is thought to represent awareness of performance. The ability to monitor, recognize, and self correct errors during and immediately following a task is important for successful everyday performance (1, 4, 5).

From a developmental perspective, children are capable of describing themselves, their personal attributes, and capabilities; however, their self-descriptions are usually positive (6). Winsler and Naglieri (7) indicate that self-awareness gradually develops during childhood, starting with awareness of concrete, attributes of behavior or physical characteristics, and graduating into more abstract attributes (8).

The emergence of metacognitive abilities that allow for recognition and differentiation between correct and incorrect performance can be evident in toddlers. Behaviors that are observed during a hide-and-seek task, such as verbalizing, peeking, or pointing to hidden objects are among the earliest signs of awareness in young children and are thought to represent strategic attempts to monitor and improve performance (3).

An increase in awareness of performance is evident around the age of 8 when children can better recognize both their positive or negative attributes (5). During late adolescence, a more integrated sense of self forms, nevertheless, there are developmental differences in the ability to accurately self appraise performance, even among typical children (9). Adolescents evaluate their performance in relation to that of others (10). They can accurately evaluate their athletic abilities or their musical performance (11, 12); however, in comparison to adults, their ability to accurately evaluate their emotional status is poorer. Studies have demonstrated that unlike adults, adolescents were less able to report their level of stress accurately, when compared to objective physiological measures (8).

Given these developmental differences in self-appraisal accuracy among typically developing adolescents, normative data are particularly important as a foundation for studying or understanding awareness deficits among adolescents with disabilities (5).

Information about the extent that typical adolescents are aware of their performance could provide a fundamental reference when assessing awareness deficits in clinical populations. Among clinical populations, such as those with neurological disability, children with ADHD, or survivors of traumatic brain injury, self-awareness deficits have been associated with lower motivation for participation in rehabilitation (13), unrealistic goals and poorer goal attainment (14, 15), less frequent use of compensatory strategies (16), and reduced safety and independent functioning (17). Steward et al. (18) reported that in relation to social and academic performance, children with ADHD were less aware of their deficits compared to typical children.

There are several quantitative methods to evaluate self-awareness. The most common strategy involves comparison of the person’s self-ratings of their function with another objective measure. Such methods evaluate whether a gap exists between two evaluations of function. These include: (1) the gap between the person’s own-ratings compared to his significant other; (2) the difference between person’s own ratings compared to the rehabilitation professionals; and (3) the gap between the person’s estimates of performance and their actual performance (19).

As described above, evaluation of awareness of performance can be achieved by calculating the discrepancy between actual and estimated performance during an activity. The adolescent version of the Weekly Calendar Planning Activity (WCPA) is an example of an evaluation that includes a measure of awareness of performance (20).

The WCPA is a performance-based measure of executive functioning that provides a broad analysis of how a person manages and copes with a complex and cognitively challenging everyday activity. The task provides information about errors and strategy use. It also compares self-estimation of performance to actual performance, as a means of providing information about the client’s awareness. This information is important in guiding intervention (21). Another valid tool by which everyday executive functions (EF) are assessed is the behavioral-rating inventory of executive functions (BRIEF) (22). This tool captures information from parents about deficits of children’s and adolescent’s parental report of everyday behaviors in their home environment, at school, and at their communities (23). Comparison between ratings made by families or rehabilitation professionals and the pe’s self-ratings could also provide a measure of self awareness (19).

To date, there are a few studies that examine awareness of performance among typical adolescents, or adolescents with learning disabilities and its relation to performance outcome. However, these studies mostly relate to academic achievements (24), handwriting performance (25), or school-related stress (26). Farrington et al. (27) highlights the importance of task awareness, strategy awareness, and performance awareness to advance the classroom academic performance. They also provide teachers with an abundance of evidence regarding the importance of metacognitive components in teaching adolescents to become learners.

However, to the best of our knowledge, among adolescents, awareness of performance has not yet been assessed in relation to “out of school” daily performance, using a performance-based assessment.

Since self-awareness could contribute to optimal task performance and may be a predictor of functional abilities and outcomes, evaluating awareness of performance in adolescents with disabilities could be particularly important in planning and guiding treatment programs. Evaluation, however, should be done in relation to the normative appropriate level of awareness. Therefore, the current study aim was to examine self-awareness of typically developed adolescents, in regard to their performance on the WCPA. We specifically aimed to answer the following questions (1) Is self-estimation of performance accuracy after the WCPA, associated with actual performance? (2) Do parent reports of adolescents EF differ in high versus low WCPA accuracy groups and in high versus low awareness groups?

We hypothesized that overestimation of performance would be associated with lower accuracy. We further hypothesized that parental reports of EF would be lower in those who overestimated performance and who were less accurate on the WCPA.

This information can contribute to understanding typical awareness of performance in adolescents and add to the normative data of the WCPA.

## Materials and Methods

### Participants

One hundred and nine healthy adolescents (31 boys and 78 girls) participated in the study, age 12–18 years, mean age = 15.2 ± 1.86 years. The participants were recruited by a “snowball,” chain-referral sampling method in which initial study participants who were acquainted with the testers subsequently referred additional participants from their own acquaintances. All participants attend the mainstream public education system in Israel, mainly from the central district in Israel. The demographic questionnaire filled by the parents was used to screen out potential participants who had neuromuscular or psychiatric conditions or education limitations that could influence the performance of independent daily activities. The questionnaire specifically asked about learning disability or other deficits that influence daily performance to make sure that any potential participant with neurological conditions will not be invited to take the test.

### Instruments

Behavior Rating Inventory of Executive Function (BRIEF) Parent Form (22) Hebrew version (28). An 86 items tool by which parents rate their child’s behavior on a three-point Likert scale (never, sometimes, and often). There are eight subscales of Initiation, working memory, plan/organize, organization of materials, monitor, inhibit, shift, emotional control, which consist the metacognition index (MI), the behavior regulation index (BRI), and the global executive composite (GEC). The higher the ratings are the greater perceived impairment they indicate. The BRIEF Parent Form was studied among clinical groups of 852 children and 1,419 control children. This yielded a normative sample and the factor analysis by which the MI and BRI were developed (22). The row scores are transformed into *T*-scores. Normative mean score is 50. Abnormal scores are indicated by *T*-scores of above 65 (29). Internal consistency ratings reported for clinical populations ranged from 0.82 to 0.98. Test–retest of 3-week correlations of clinical populations ranged from 0.72 to 0.84. The BRIEF—Hebrew version showed a moderate-high internal reliability and reviled significant differences for EF between typical children and children with ADHD (28).

In the current study, the three main index scores (GEC, BRI, and MI) were used.

Weekly calendar planning activity (20) is a performance measure of executive function by which the participant is presented with a randomly ordered list of 18 appointments. He is asked to enter the appointments into a 1-week schedule. The task consists of conflicts and five written rules: (1) leave Wednesday free, (2) do not cross out appointments once they are entered, (3) inform the examiner of specified time, (4) ignore distracting questions from the examiner, and (5) inform the examiner when finished. The participants need to recognize and manage them while entering the appointments. The examiner observes and records the strategies used by the participants during the task from a list of 13 pre-identified strategies. The scores indicate the number of accurate appointments, errors made in appointment placement, the planning times and the total time of completion, the number of rules followed, the types of strategies used and the efficiency score. The task is followed by eight after task questions. In the current study, the after-task question that was examined was “*Estimate the number of appointments that you entered accurately into the schedule*.” This question was used for assessing awareness of accuracy of performance. This estimated judgment of accuracy was compared to actual accuracy scores the discrepancy between them was used to measure awareness to performance.

Cross-cultural normative data for the adult version as well as data on typical adult performance across three age groups was provided by Toglia et al. (30). Accuracy scores were similar across cultures. People who were aged 65 years and above were less accurate and strategic compared to younger adults (*p* < 0.05), regardless of culture. Israeli younger groups were more strategic, slower, and less efficient (*p* < 0.05) than Americans.

The WCPA University student version was found to reliably distinguish between students with and without ADHD. Students with ADHD used significantly less strategies, took more time, and were less accurate than those without ADHD (31). Similarly, the WCPA youth version was found to distinguish between at-risk youth aged 16–21 years and typical community high school students (21). The at-risk group made more errors, used fewer strategies, and broke more rules than the community group. The WCPA youth version was found to be useful in identifying adolescents who are at risk for functional performance deficits. Although research has examined strategy use and accuracy of the WCPA, awareness of performance as measured by the difference between actual and estimated accuracy on the WCPA has not yet been studied.

To date, there are no published data for ages 12–16 years in using the WCPA.

In the present study, the WCPA accuracy estimation was compared to other WCPA scores including the completion total time, number of accurate meetings, number of strategies, number of rules, and efficiency scores.

### Procedures

Ethical approval was received from the Institutional Review Board of the University (no. 297/15). Data collection was performed by third year Occupational therapy students who were trained in administrating the assessment. The study session was a onetime meeting with the examiner. After both the parents and the adolescents signed the consent form, the participants filled the demographic questionnaire and then performed the WCPA. Their parents filled the BRIEF in a separate quiet room at their home.

The participants were divided into two groups according to their accuracy scores. The WCPA accuracy median score was 14.0, mean = 13.92; SD = 2.4. Those who had more than 14 accurate number of meetings were assigned to the high accuracy group and those who had 14 or less than 14 accurate meetings were assigned to the low accuracy group. The WCPA measures and BRIEF scores were then compared between the high and low accuracy groups.

Awareness was measured by calculating the discrepancy between the actual number of accurate meetings (WCPA accuracy score) and the adolescents’ self-estimation of their accuracy score. This created the awareness accuracy score. Also, those who had no discrepancies (0 or 1) or underestimated the number of meetings (indicated noticing a lot of errors) were assigned to the good awareness group. Those who overestimated their scores by 2 or more meetings were considered to have poor awareness and were assigned to the overestimation group. The WCPA measures were then compared between both awareness groups.

### Data Analysis

Descriptive statistics (means, medians, SDs, skewness, kurtosis, and ranges) were calculated for all variables. The efficiency score, total time, estimation of accuracy, and the number of entered meetings were not normally distributed, thus, the non-parametric Mann–Whitney *U*-test was used to measure differences between the groups. *T*-test was used to measure age differences. A multivariate analysis of variance was used to examine whether differences in the tested variables exist between genders. One-way ANOVA was used to examine whether differences exist between age groups (12–13, 14–15, 16–18 years) in all the tested variables. Correlations were also measured between the actual performance and the awareness of performance using Pearson. With Bonferroni correction, the level of significance was set at *p* ≤ 0.01.

## Results

No significant age differences were found. Age did not correlate with any of the study variables (*p* > 0.01).

Gender differences were identified only for overestimation of performance and not for other variables. Overestimation of performance was significantly more frequent among boys [*F*_(107)_ = 6.08, *p* = 0.015] Therefore, correlations and comparisons of the awareness of accuracy score was calculated for boys and girls separately.

In order to present an elaborated normative data, Table 1 is presented for three age groups (age 12 years to 12 years and 11 months; age 13 years to 14 years and 11 months; 16–18 years). WCPA subscales and the BRIEF sub- scales means, medians, SDs, skewness, kurtosis, and ranges.

**Table 1:** The weekly calendar planning activity (WCPA) and BRIEF subscales scores (*N* = 109) across three age groups.

	WCPA								BRIEF		

Age 12–13 years and 11 months (*n* = 32)											

	Total time (s)	Accurate meetings	Number of entered meetings	Number of strategies	Efficiency	Estimation of accuracy	Awareness of accuracy	Awareness of accuracy	Metacognition index (MI)	BRI	Global executive composite (GEC)

							Girls (*n* = 26)	Boys (*n* = 6)			
Mean	1,927.09	13.43	18.00	4.81	192.84	15.59	1.88	3.33	48.09	50.81	47.71
Median	1,756.50	13.00	18.00	4.00	178.11	16.00	1.50	3.50	48.00	49.00	46.50
SD	835.07	2.57	0.00	2.64	85.86	2.79	3.30	1.75	6.92	9.32	8.79
Skewness	0.98	0.22		0.78	0.96	−1.97	−0.41	0.24	0.40	1.52	1.21
Kurtosis	0.56	−0.89		0.29	0.79	3.74	−0.35	−0.01	0.00	3.79	3.02
Minimum	743.0	9.00	18.00	0.00	78.90	7.00	−6.00	1.00	37.00	35.00	36.00
Maximum	4,080.0	18.00	18.00	11.00	438.70	18.00	6.00	6.00	66.00	83.00	78.00

**Age 14–15 years and 11 months (*n* = 36)**											

	**Total time (s)**	**Accurate meetings**	**Number of entered meetings**	**Number of strategies**	**Efficiency**	**Estimation of accuracy**	**Awareness of accuracy**	**Awareness of accuracy**	**MI**	**BRI**	**GEC**

							**Girls (*n* = 27)**	**Boys (*n* = 9)**			
Mean	1,770.21	14.05	17.97	4.72	168.35	16.41	2.25	2.66	47.16	49.25	46.94
Median	1,497.50	14.00	18.00	4.00	143.87	17.00	2.00	3.00	48.00	48.50	48.00
SD	911.87	2.25	0.16	2.76	98.03	1.79	2.50	1.73	6.70	7.53	7.79
Skewness	0.94	−0.54	−6.00	0.74	1.40	−1.77	0.11	−0.26	0.38	0.63	0.32
Kurtosis	0.17	−0.27	36.00	−0.34	1.51	4.02	1.66	−1.43	−0.33	−0.05	−0.68
Minimum	286.0	9.00	17.00	1.00	53.00	10.00	−4.00	0.00	35.00	37.00	35.00
Maximum	3,900.0	17.00	18.00	11.00	426.66	18.00	9.00	5.00	64.00	69.00	63.00

**Age 16–18 years (*n* = 41)**											

	**Total time (s)**	**Accurate meetings**	**Number of entered meetings**	**Number of strategies**	**Efficiency**	**Estimation of accuracy**	**Awareness of accuracy**	**Awareness of accuracy**	**MI**	**BRI**	**GEC**

							**Girls (*n* = 25)**	**Boys (*n* = 16)**			
Mean	1,550.12	14.21	18.00	5.07	141.33	16.36	1.24	3.56	46.09	48.73	46.65
Median	1,200.00	14.00	18.00	5.00	127.33	17.00	2.00	2.50	46.00	48.00	46.00
SD	865.88	2.38	0.22	2.64	68.86	2.28	3.03	2.65	7.58	7.97	7.47
Skewness	1.60	−0.82	0.00	0.44	1.05	−2.87	−1.10	1.12	0.50	0.43	0.47
Kurtosis	2.15	1.15	20.00	−0.33	0.77	10.40	2.50	0.830	−0.41	−0.64	−0.14
Minimum	573.0	7.00	17.00	0.00	51.06	6.00	−8.00	0.00	35.00	37.00	35.00
Maximum	4,273.0	18.00	19.00	11.00	333.33	18.00	6.00	10.00	64.00	66.00	66.00

As can be evident in Table 1: as a group, adolescents entered an average of 18 appointments, among these, 13.93 (SD = 2.40) appointments were accurate. The mean efficiency score was 165.3 (SD = 1.2) with a mean total time of 1,733.5 (SD = 878.4) and estimation of accuracy of 16.1 (SD = 2.3) appointments. These scores were not normally distributed. The median overestimation of the group was 2. Participants used 4.9 strategies (SD = 2.66) on average. Also, the average BRIEF scales scores among the participants across all age groups were within the normative average of the BRIEF for each of the sub scales (*T* < 65).

### Correlations between Actual Performance and Awareness of Performance

Significant correlations were found between the WCPA accuracy score and the level of awareness of performance. Among girls [(*n* = 78), *M* = 1.80, SD = 2.95, *r*_s_ = −0.66, *p* = 0.00] and among boys [(*n* = 31), *M* = 3.25, SD = 2.23, *r*_s_ = −0.86, *p* = 0.00]. Higher awareness was significantly associated with better accuracy of performance.

**Figure 1:**
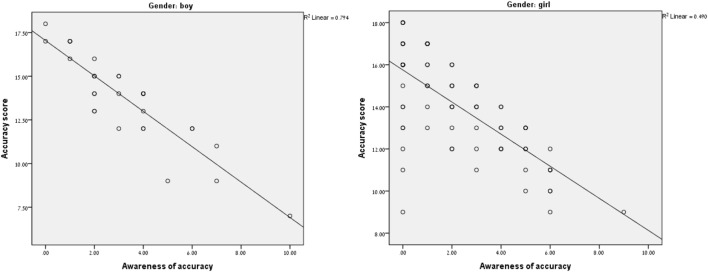
Correlations between the Weekly Calendar Planning Activity awareness score and the accuracy score for girls (*n* = 78) and boys (*n* = 31).

As illustrated in Figure 1: a significant medium (*R*^2^ = 0.49) linear relationship was found between awareness and accuracy among the girls, and a significant strong (*R*^2^ = 0.79) linear relationship among the boys. Participants with higher accuracy scores demonstrated higher awareness.

Table 2 illustrates that the high accuracy group had significantly better parental rating of EF across in the BRIEF MI, BRI, and GEC subscales (*p* < 0.01) specifically in initiation (*Z* = −2.94, *p* < 0.01), working memory (*Z* = −3.46 *p* < 0.01), planning and organizing (*Z* = −3.17 *p* < 0.01), and monitoring (*Z* = −2.80 *p* < 0.01). Also near significance in emotional control (*Z* = −1.98 *p* = 0.04) and shifting (*Z* = −2.26 *p* = 0.02).

**Table 2:** Comparison between high and low weekly calendar planning activity (WCPA) accuracy groups on parental reports of executive functions (EF) (BRIEF) and other WCPA measures.

		High accuracy >14			Low accuracy < 14			Mann–Whitney-*U*/*T* test
		*n* = 47			(*n* = 62)			
		Girls (35) Boys (12)			Girls (43) Boys (19)			

		*M*	Med	SD	*M*	Med	SD	*Z*/*t*
Age		15.23	15	1.85	14.84	15	1.86	*t* = −1.08 NS
	Number of accurate meetings	16.14	16	1.02	12.25	12.50	1.66	*Z* = −15.02^a^
	Awareness of accuracy (girls)	0.51	1	1.61	2.86	4	3.36	*Z* = −4.59^a^
	Awareness of accuracy (boys)	1.50	1	1.50	4.36	4.00	2.08	*Z* = −3.96^a^
	Efficiency score	132.09	113.76	62.44	190.61	169.85	93.37	*Z* = 3.91^a^
	Total time	1,919.9	1,680.0	978.50	1,592.14	1,130.0	772.70	*Z* = −1.89 NS
	Number of rules followed	4.55	5.00	0.58	4.08	4.0	0.96	*Z* = −3.17^a^
	Number of strategies	5.36	4.0	3.06	4.51	4.0	2.281	*Z* = −1.65 NS

BRIEF	BRI	46.95	46.0	7.22	51.45	48.0	8.45	*Z* = 2.92^a^
	Metacognition Index	43.78	42.0	6.72	49.17	50.0	8.11	*Z* = 3.69^a^
	Global Executive Composite	44.34	44.0	5.97	49.45	49.0	7.00	*Z* = 4.01^a^

*^a^>0.01*.

*M, mean; Med, median*.

Significantly better performance was indicated in the majority of WCPA subscales (*p* < 0.01). The higher accuracy group was more efficient (*p* < 0.01), followed more rules (*p* < 0.01) and had higher awareness of their performance (*p* < 0.01) for both genders. No significant difference was found in the number of strategies used or the total time required.

Table 3 indicates that participants of both genders in the good awareness group were significantly more accurate in performing the WCPA (*p* < 0.01). The girls in the good awareness group were significantly more effective (*p* < 0.01), then those of the overestimation group and also had significant better parental rating on the BRIEF GEC subscale (*p* < 0.01) and MI, sub scale (*p* < 0.01). As significant level was set to *p* < 0.01, near significance was found in initiation (*Z* = −2.29, *p* = 0.02) and monitor (*Z* = −2.10, *p* = 0.03) subscales. No significant difference was found in the BRI subscale and in the WCPA number of strategies used, number of rules followed, or total time required. Among the boys, near significance was found for number of rules followed (*p* = 0.04) and total time required (*p* = 0.02). No significant difference was found in the BRIEF subscales and in the WCPA number of strategies used.

**Table 3:** Comparison of weekly calendar planning activity (WCPA) and BRIEF scores between groups with high and low awareness.

		*M*	Med	SD	*M*	Med	SD	*Z*/*t*
		
Girls		Good awareness of accuracy (*n* = 43)			Overestimation of accuracy (*n* = 35)			Mann–Whitney-*U*/*T*-test
Age		15.06	15	1.97	14.44	14	1.60	*t* = 1.50 NS

BRIEF	BRI	48.34	46	6.54	50.65	50	7.64	*Z* = −1.65 NS
	Metacognition index (MI)	45.11	45	9.09	50.34	50	8.96	*Z* = −2.65^a^
	Global Executive Composite (GEC)	46.04	45	6.52	49.54	51	6.95	*Z* = −2.50^a^

WCPA	Awareness of accuracy	0.18	1.00	2.02	3.80	4	2.68	*Z* = −6.37^a^
	Number of accurate meetings	15.27	16.00	1.96	12.45	12	1.80	*Z* = −5.14^a^
	Efficiency score	141.15	127.77	75.31	197.23	186.00	88.86	*Z* = −5.41^a^
	Total time	1,841.57	1,639.00	970.06	1,726.39	1,380.00	873.81	*Z* = −0.56 NS
	Number of rules followed	4.48	0.668	1,380.0	4.08	4	0.98	*Z* = −1.78 NS
	Number of strategies	5	4	2.83	4.97	4	2.44	*Z* = −1.32 NS

**Boys**		**High awareness of accuracy (*n* = 14)**			**Overestimation of accuracy (*n* = 17)**			**Mann–Whitney-*U*/*T*-test**

Age		15.78	17	1.92	15.5	15	1.81	*t* = 0.52 NS

BRIEF	BRI	49.28	49.50	6.46	50.29	49.0	8.60	−0.119 NS
	MI	44.42	41.50	8.66	46.05	46.0	6.721	−0.795 NS
	GEC	45.07	43.50	7.60	47.35	46.0	7.23	−0.916 NS

WCPA	Awareness of accuracy	1.42	2	0.75	4.76	4	1.88	−4.803^a^
	Number of accurate meetings	15.50	15.50	1.60	12.29	12	2.25	−3.694^a^
	Efficiency score	141.73	141.15	59.52	180.53	135.0	105.33	−0.794 NS
	Total time	1,851.85	1,521.0	822.24	1,377.17	1,080.00	622.81	−2.204^b^
	Number of rules followed	4.5714	5	0.75593	3.9412	4	0.89935	−2.239^b^
	Number of strategies	5.07	5	2.70226	4.2353	4	2.77330	−0.723 NS

*^a^>0.01*.

*^b^>0.05*.

*M, mean; Med, median*.

## Discussion

The goal of the current study was to examine self-awareness of typically developed adolescents, immediately following their performance on the WCPA, a multi-level everyday calendar task. We specifically examined the association between self-estimation of performance accuracy after a task compared to actual performance. We also compared parental reports of adolescents EF between high versus low WCPA accuracy groups and high versus low awareness groups. Additionally, our results contribute to establishing a normative baseline for typical adolescent WCPA performance that allows for comparison with adolescents with disabilities or those who are at risk for decreased self-awareness and everyday executive function deficits.

### Self-Awareness of Performance

The results of the study revealed that participants who did better on the WCPA and had high accuracy scores were more likely to show high awareness of performance, compared to participants with low accuracy scores. The high accuracy group had also better parental ratings on the BRIEF subscales, and they were more efficient in performing the WCPA, followed more rules and had better awareness of their performance. To conclude, higher awareness in the present study was associated with higher performance accuracy for both genders.

The association we found between awareness and performance was supported by Schoo et al. (32) who measured self-awareness of EF performance among typical young and older adults. They indicated that the poorer the competence in a certain capacity, the larger the overestimation. They attributed this association to the universal phenomenon that is known as the “Dunning–Kruger effect” (33). The Dunning–Kruger effect indicates that a person with low ability is less likely to objectively evaluate his actual competence or incompetence. This is consistent with our findings in which individuals with good awareness were indeed more likely to show high accuracy and vice versa.

In the present study, overestimation was more prevalent among the boys.

The psychological literature offers explanations to this phenomenon in which boys tend to overestimate performance more than girls. Beyer and Bowden (34) suggested that gender differences in self-evaluation are mediated by expectancies, and also by the way people present themselves. The authors indicated that, in unfamiliar tasks, women try to appear modest and lower expectations of their performance, whereas man tend to overestimate performance, show more confidence, and present themselves in a more positive and abled way.

This phenomenon of overestimation can be evident even after performance, for which, the gender bias expectancies mediated self-evaluation even more than actual feedback. Bayer found that, even in task where feedback on performance could be obtained and allow for an accurate evaluation, men’s overconfidence led to a more positive self-evaluation, and an opposite pattern was observed in women’s self-evaluations who underestimated their performance. However, since feedback on performance could not easily be obtained when performing the WCPA, the extent to which self-efficacy or self-confidence do contributed to the estimation of performance in relation to the actual performance cannot be clearly stated and should be further explored with a larger sample, looking also on self-efficacy or self-confidence.

### Association between Parental Reports of EF and WCPA Performance

Our results in which better awareness was associated with better daily EF performance as reported by parents, are consistent with the recent study of Steward et al. (18), who measured awareness and EF performance of adolescents with and without ADHD. Using the BRIEF-SR, a self-report measure compared to the parents’ report, similar to our study, they found that better awareness was associated with better daily EF performance as reported by parents.

Also, higher performance accuracy for both genders was associated in our study with better daily performance as reported by parental reports of EF, especially regarding the working memory, initiation, planning, organizing and monitoring.

### BRIEF Subscales

Lower working memory could contribute to difficulty in recognizing or monitoring errors in performance, leading to lower accuracy. This is noted by Unsworth (35) and Unsworth et al. (36) who indicated that working memory and self-initiation of cues were associated with better performance accuracy in a long-term memory task. Individuals with better working memory capacity were also found to be more effective in self-generating retrieval cues and more effectively monitored performance (37). Although, as expected, there was no gender difference in the BRIEF scores, we found that girls in the good awareness group had significantly better parental rating of EF on the GEC and MI sub scales, yet, this difference was not found for boys.

It could be that among girls, parents are more sensitive to behaviors indicated in the MI subscale, whereas these behaviors are less distinguishable among typical boys. Jensen et al. (38) indicated, for instance, bias in parental report that is based on gender and adult expectations, in which aggression in a girl may be less tolerated and more widely reported than aggression in a boy. The gender differences in the parental rating could also be mediated by the gender of both the parent and the child, and could influence parents’ perceptions. van der Veen-Mulders et al. (39) investigated parent agreement on rating children’s externalizing behavior problems, both in a clinical and a nonclinical sample. They suggested that discrepancy in parent reports of behavioral problems may reflect differences in the parents own perspectives regarding the same behaviors. Not only that mothers and fathers may observe their children in different contexts but also the time spent with the child could influence their reports on the child’s behavior.

Thus, information on the parents themselves could help in interpreting our results in respect to gender differences. Unfortunately, an explanation for what may mediate parent’s reports on their child’s performance in relation to gender cannot be determined from the present research, as information on whether mothers or fathers filled the BRIEF was not provided, as well as more information of their beliefs and attitudes. This could, however, raise new questions for future studies.

### Limitations and Recommendations

The current study used a relatively small sample size that was largely homogeneous in terms of ethnicity. The lower number of boys also limit comparison between genders. Since socioeconomic status were not considered in the analyses, future researchers should seek to include a more diverse sample in terms of socioeconomic status, gender and ethnicity, and a larger sample. Further exploration of gender differences in self-evaluation and possible mediating factors such as self-confidence or self-efficacy is recommended. Also, the extent to which overestimation of abilities and EF impairments overlap should be examined in future studies with this population.

Inclusion criteria were based on parental responses to a questionnaire; therefore, we cannot rule out the possibility that a participant had an undiagnosed or undisclosed condition such as ADHD. Our results, therefore, should be interpreted with caution.

## Conclusion

Awareness of performance is a building block required to achieve successful outcomes in daily life. As stated by Toglia and Kirk (4), self-awareness should be assessed within the context of actual performance. Supported by parental reports, self-estimation of performance as measured immediately following the WCPA task, provided valuable quantitative information. The results of this study contribute to inclusion of normative data on estimation of performance for adolescent performance on the WCPA assessment and in relation to each gender. This information provides a foundation for assessing and comparing self-awareness in adolescents with disabilities to that of typical adolscents.

Self-appraisal immediately following task performance may provide valuable information for treatment planning. Adolescents self-evaluation of performance in the current study was associated with their level of actual performance. This implies that performance deficits might be improved by addressing awareness of one’s performance. Toglia and Kirk (4) suggest that methods to enhance self-awareness should aim toward helping people self-discover their own errors. Adolescents that are less aware of their errors in performance might benefit from metacognitive training techniques, including strategies that focus on self-monitoring skills and guided questions to help them recognize their own errors. This could support adolescent functional performance across a wide range of everyday tasks and contexts. Clinicians are encouraged to integrate awareness assessment into everyday clinical practice and research on adolescents. This study is the first to include a large number of typical adolescents that are less than age 16 years and adds to the normative data and feasibility of using the WCPA with this younger group.

## Ethics Statement

Ethical approval was received from the Institutional Review Board of the University of Haifa (no. 297/15).

## Author Contributions

As the corresponding author, SZ was in charge of the research examiners team, supervised the data collection process, and data analysis, established the initial draft of this manuscript, and refined it as a mutual process with JT, who provided great contribution to the literature review, the data interpretation, and conclusion, as well as to the American English proof and style.

## Conflict of Interest Statement

The authors declare that the research was conducted in the absence of any commercial or financial relationships that could be construed as a potential conflict of interest.
